# *In vitro* cellular tropism and immunomodulatory response to rVSVΔG-ZEBOV-GP in human cells derived from tissues associated with adverse events

**DOI:** 10.1128/spectrum.00408-25

**Published:** 2026-03-24

**Authors:** Paola Martinez-Murillo, Catia Alvarez, Francesco Santoro, Maria Novedrati, Chiara Sonnati, Giorgio Montesi, Simone Lucchesi, Donata Medaglini, Isabella Eckerle, Claire-Anne Siegrist, On behalf of the VEBCON, VSV-EBOVAC, and VSV-EBOPLUS Consortia

**Affiliations:** 1 Center of Vaccinology, Department of Pathology and Immunology, Faculty of Medicine, University of Geneva 27212, Geneva, Switzerland; 2 Department of Medicine, Faculty of Medicine, University of Geneva 27212, Geneva, Switzerland; 3 Department of Medical Biotechnologies, University of Siena 9313, Siena, Italy; 4 Geneva Centre for Emerging Viral Diseases, Geneva University Hospitals 27230 , Geneva, Switzerland; 5 University of Geneva Medical School, Geneva, Switzerland; Universidade Federal do Rio de Janeiro, Rio de Janeiro, Brazil

**Keywords:** rVSVΔG-ZEBOV-GP, adverse events, vaccination, innate response

## Abstract

The recombinant vesicular stomatitis virus–vectored Zaire Ebola virus glycoprotein (rVSVΔG-ZEBOV-GP) vaccine, while effective and well-tolerated, exhibits notable reactogenicity, manifesting in expected adverse events (AEs), such as fever, headache, and pain, along with rare, unexpected AEs, including skin lesions, cutaneous vasculitis, and transient arthritis. The presence or absence of AEs following rVSVΔG-ZEBOV-GP vaccination is associated with a specific innate plasma signature. This study aims to elucidate *in vitro* the tropism of the vaccine for different cell types derived from tissues previously reported to be involved in the unexpected AEs. Upon *in vitro* infection with rVSVΔG-ZEBOV-GP, various cell types, such as synoviocytes, fibroblasts, keratinocytes, and endothelial cells (except chondrocytes), demonstrate productive infection, which in dermal fibroblasts triggered the release of many innate plasma signature markers, including keratinocytes’ pro-inflammatory and proapoptotic cytokines such as OSM and TRAIL. Infected monocytes from buffy coats, activated by infection, produce most innate plasma signature markers. In co-culture, rVSVΔG-ZEBOV-GP-infected monocytes serve as a source to synoviocyte infection, resulting in distinct kinetics modulation in innate biomarkers (transcription and secretion) and upregulation of specific genes, such as *NEDD8* and *SIGLEC-1*, which have been associated with inflammatory arthritis in animal models. Altogether, our work, based on *in vitro* studies, provides insights into the possible mechanisms of rVSVΔG-ZEBOV-GP underlying the observed reactogenicity by showing tropism of the vaccine for off-target cells derived from AE-affected compartments (skin, joints, vessels). Furthermore, *in vitro* interaction with infected monocytes modulates the innate response of synoviocytes.

**IMPORTANCE:**

Our study expanded knowledge about the cellular tropism of rVSVΔG-ZEBOV-GP vaccine toward peripheral blood mononuclear cells and cell lines derived from tissues (skin, vessels, and joints) associated with unexpected AEs, as well as their possible contribution to the vaccine-induced innate response. Using *in vitro* infection and co-culture techniques, we showed that rVSVΔG-ZEBOV-GP-infected monocytes can transmit the virus to synoviocytes, and how infection affects human monocytes and synoviocytes at the protein and transcriptomic levels. Our findings provide insights into the *in vitro* off-target infection dynamics and innate immune response triggered by rVSVΔG-ZEBOV-GP vaccine. While these results may enhance understanding of rVSV-based vectors, *in vivo* relevance remains unclear, as does whether the effects come from the VSV backbone or Ebola GP. These findings support the evaluation of off-target effects of rVSV-based vaccine candidates, including those under development for hemorrhagic fever viruses, such as Marburg virus, Sudan virus, and Lassa virus, especially if similar AEs are observed.

## INTRODUCTION

Ebola virus (EBOV) is the cause of a severe viral hemorrhagic disease of zoonotic origin. Human-to-human transmission of the virus occurs through direct contact with blood, secretions, or other bodily fluids of infected individuals (1). Ebola virus disease (EVD) is endemic to Central and Western Africa (1, 2). Since its discovery in 1976, EBOV has caused geographically limited outbreaks with several to a few hundred cases; however, two large outbreaks have occurred in West Africa (2014–2016) and the Democratic Republic of Congo (2018–2020), driving both interest in the disease and the development of vaccines and therapeutics (1).

The rVSVΔG-ZEBOV-GP vaccine is a replication-competent, live-attenuated, recombinant vesicular stomatitis virus vaccine (3, 4) that has been genetically engineered to express the gene of the glycoprotein from the Zaire ebolavirus, replacing the gene of the native VSV glycoprotein (5, 6). Initial ring vaccination efficacy trials indicated that the rVSVΔG-ZEBOV-GP vaccine has high efficacy (100% in a controlled environment) (7, 8). Recent real-world data confirms that a single dose of rVSV-ZEBOV is highly protective against EVD 10 days or more after vaccination (84% [95% credible interval 70–92]) and therefore remains a critical tool for outbreak response to EBOV (9).

The rVSVΔG-ZEBOV-GP vaccine has been shown to be safe and immunogenic, but reactogenic (5, 7, 10, 11). Unexpected adverse events (AEs), including transient arthritis, vesicular lesions, and cutaneous vasculitis, occurred during the first two to three weeks after vaccination in a proportion of vaccinees participating in two clinical trials (5, 11). In the Geneva phase I clinical trial, transient arthritis was observed in 24% of vaccinees with a median of 11 days after vaccination; joints were affected asymmetrically by swelling, tenosynovitis, and bursitis (11). These results were replicated in another study, in which the onset of arthritis occurred at the same median time and duration but at a lower rate (5%) (12). In addition, in some of these cases, rVSVΔG-ZEBOV-GP RNA was detected by qRT-PCR in synovial fluid, vesicles, and in skin lesions a few weeks after vaccination, suggesting rather a direct effect on these compartments rather than a solely immune-mediated etiology (5, 11, 12). rVSVΔG-ZEBOV-GP viremia was common but transient and dose-dependent, and viremia did not correlate with the transient arthritis observed (5). These findings suggest that, in some patients, components of the vaccine virus may persist in immune-privileged sites despite an intact innate immune response. Importantly, because all testing was performed using RT-PCR, which identifies viral RNA but cannot distinguish between infectious virus and degraded genetic material. It remains uncertain whether these findings reflect persistence of replication-competent VSV-ZEBOV or simply residual, non-infectious RNA fragments. In this context, reinforcing the role of the innate response, we have recently reported a monocyte innate plasma vaccine signature that correlates with viremia, AEs, and hematological changes (13, 14).

To better understand the cell and tissue tropism of this vaccine, it is crucial to evaluate the target cells and body compartments affected by expected and unexpected AEs. Given the successful implementation of the rVSVΔG-ZEBOV-GP vaccine, it is expected that other rVSV-derived vaccines for emerging viruses would be developed in the future; therefore, investigating the pathophysiological mechanism behind these unexpected AEs observed with the rVSVΔG-ZEBOV-GP is relevant, in case similar events arise with new rVSV-based vaccine candidates. In this study, we have investigated the *in vitro* cellular tropism (off-target infection and replication) and the production of innate immune biomarkers after rVSVΔG-ZEBOV-GP infection in a panel of human primary cells from tissues previously associated with unexpected AEs, as well as peripheral blood mononuclear cells (PBMCs). We also set up co-culture assays of monocytes and synoviocytes to assess whether monocytes could be the source of synoviocyte infection and to study *in vitro* the kinetics of the response at the protein and transcriptomic levels.

## MATERIALS AND METHODS

### Healthy PBMCs and CD14+ monocyte isolation

We isolated healthy PBMCs and CD14+ monocytes from buffy coats. PBMCs were isolated by density-gradient centrifugation from ethylenediaminetetraacetic acid (EDTA) buffy coats using Ficoll-Paque PLUS (GE Healthcare), washed extensively in phosphate-buffered saline (PBS), and treated with red blood cell lysis buffer. Cells were counted and frozen in 90% heat-inactivated fetal bovine serum (FBS) and 10% dimethyl sulfoxide (DMSO) (Sigma-Aldrich). CD14+ monocytes were recovered from freshly isolated PBMCs using the EasySep Human Monocyte Isolation Kit (Stemcell Technologies).

### Cell culture of immortalized and primary cells

We used immortalized cell lines and primary cells (Table S1). The immortalized cell lines—Monkey kidney cells (Vero E6) and human synovial sarcoma cells (SW982)—were cultured in Dulbecco’s Modified Eagle Medium (DMEM) and GlutaMAX (Gibco, Thermo Fisher Scientific) supplemented with 50 mL FBS, 5 mL non-essential amino acids, and 5 mL penicillin-streptomycin. The primary cells (human knee articular chondrocytes) (NHAC-Kn, Lonza) were cultured in chondrocyte basal medium (Lonza) supplemented with 25 mL FBS, 0.5 mL GA-1000, 2.5 mL bFGF, 1 mL R3-IGF, 0.5 mL transferrin, and 1 mL insulin. Human synoviocytes (HS, ScienCell) were cultured in synoviocyte medium (SM, ScienCell) supplemented with 10 mL FBS, 5 mL synoviocyte growth supplement (SGS), and 5 mL penicillin-streptomycin. Human dermal fibroblasts (NHDF-Ad, Lonza) were cultured in fibroblast growth basal medium (FBM, Lonza) supplemented with 10 mL FBS, 0.5 mL rhFGF-B, 0.5 mL GA-1000, and 0.5 mL insulin. Human epidermal keratinocytes (NHEK-Ad, LZ-192627, Lonza) were cultured in keratinocyte cell basal medium (KBM-Gold, Lonza) supplemented with 0.5 mL hydrocortisone, 0.5 mL transferrin, 0.25 mL epinephrine, 0.5 mL GA-1000, 2 mL BPE, 0.5 mL hEGF, and 0.5 mL insulin. Human dermal microvascular endothelial (HDMEC, ScienCell) were cultured in endothelial cell medium (ECM, ScienCell) supplemented with 25 mL FBS, 5 mL ECGS, and 5 mL penicillin-streptomycin. Human dermal lymphatic endothelial (HDLEC, Cell Biologics) were cultured in human ECM (Cell Biologics) supplemented with 25 mL FBS, 0.5 mL VEGF, 0.5 mL heparin, 0.5 mL EGF and FGF, 0.5 mL hydrocortisone, 5 mL L-glutamine, and 5 mL penicillin-streptomycin. Human umbilical vein endothelium cells (HUVEC, Lonza) were cultured in endothelial cell growth medium (EGM, Lonza) supplemented with 10 mL FBS, 0.5 mL GA-1000, 2 mL hFGF-B, 0.2 mL hydrocortisone, 0.5 mL R3-IGF-1, 0.5 mL VEGF, 0.5 mL ascorbic acid, 0.5 mL hEGF, and 0.5 mL heparin. Cells were passaged when confluent and grown at 37°C with 5% CO_2_.

### rVSVΔG-ZEBOV-GP vaccine stock

The rVSVΔG-ZEBOV-GP vaccine was used to infect fresh confluent VeroE6 cells for 24 h. The next day, the supernatant was collected and concentrated by centrifugation in a Vivaspin column (Sigma-Aldrich) for 30 min at 3,000 × *g*. Aliquots were stored at −80°C, and the stock was titrated by plaque assay.

### Viral titration by plaque assay

The plaque assay was performed in duplicate infecting Vero E6 cells with serial dilutions (from 10^−1^ to 10^−6^) of the viral stock. Cells were washed twice and covered with a 1:1 mixture of 2× DMEM medium (Gibco) and 2.4% Avicell (Dupont) to prevent the virus infection from spreading indiscriminately. Infected cells were fixed 24 h post-infection with 4% paraformaldehyde (PAF) for 30 min at room temperature (RT) and stained with crystal violet (Sigma-Aldrich) for 30 min at RT. Viral plaques were counted manually. The plaque counts, together with the dilution factor, were used to calculate the number of plaque-forming units per sample unit volume (PFU/mL).

### rVSVΔG-ZEBOV-GP infection of cell lines and PBMCs

We infected primary human cells and cell lines derived from the respective compartments (Table S1). Primary and immortalized cells were plated in a 24-well plate at a density of 3 × 10^5^ cells per well, while thawed PBMCs were plated in round-bottom 96-well untreated polystyrene Corning plates at a density of 2 × 10^6^ cells per well. Cell lines were infected the next day, while PBMCs were infected after thawing. Infection with rVSVΔG-ZEBOV-GP was done in serum-free medium at different multiplicity of infection (MOI), with mock controls in parallel. For PBMCs and purified monocytes, we used an MOI of 1 to maximize infection efficiency in this heterogeneous population and to allow detection by FACS. For primary stromal cells (synoviocytes, fibroblasts, endothelial cells), we used an MOI of 0.1 to evaluate potential off-target infection under lower viral pressure, reflecting their lower permissiveness and to better mimic conditions relevant to *in vivo* exposure. After incubation for 1 h at 37°C, cells were extensively washed, fresh growth medium was added, and the supernatant was collected to quantify viral RNA by RT-qPCR at multiple time points post-infection. T0 samples were included as baseline controls to capture residual input virus.

### RNA extraction and real-time quantitative polymerase chain reaction (RT-qPCR)

RNA was extracted from infected cells using the NucliSens easyMAG (BioMérieux), according to the manufacturer’s instruction. The viral load was determined from RNA by RT-qPCR using the SuperScript III Platinum One-Step qRT-PCR Kit (Invitrogen) in CFX96 Thermal Cycler (Bio-Rad Laboratories). The RT-qPCR was performed using a specific set of primers (forward: 5′-CGGAGGATTGACGACTAATGC-3′, Microsynth; reverse: 5′-CGAGCCATTCGACCACATC-3′, Microsynth) and probe (5′-CGCCACAAGGCAG-3′, Microsynth).

### Western blot of Ebola receptors expression

We looked at two different receptors: T-cell immunoglobulin and mucin domain 1 (TIM-1), which is present on the cell surface and serves as a receptor/cofactor for EBOV entry *in vivo*, enhancing viremia and pathogenesis (15, 16), and Niemann-Pick C1 (NPC1), an intracellular lysosomal cholesterol transporter required for Ebola RNA release through endosomal membrane (17–19).

Cells were lysed with lysis buffer (NP-40) containing a protease inhibitor (Roche). Samples were sonicated and centrifuged 10 min at 20,000 × *g* at 4°C, then incubated for 5 min at 95°C. Protein lysates (10 μL for immortalized cells and 20 μL for primary cells) were loaded onto a 10% polyacrylamide gel and run at 150 V for 2 h. Transfer was done on a PVDF membrane at 150 mA for 1 h and blocked with 5% milk in washing buffer for 30 min. The membrane was incubated with anti-NPC1 antibody (rabbit, Abcam) or anti-TIM-1 antibody (mouse, MAB1750-100, Bio-Techne AG) overnight at 4°C (dilutions 1:2,000, 1 μg/mL respectively). After washing, the membranes were incubated with the secondary antibodies—goat anti-rabbit IgG HRP-conjugate (Bio-Rad Laboratories) or goat anti-mouse IgG HRP-conjugate (Bio-Rad Laboratories) (dilution 1:5,000)—for 1 h at RT. After washing, the membrane was developed using the ECL system (Advansta), and pictures were taken. Membranes were re-incubated with an HRP anti-beta actin antibody (Abcam) for 1 h at RT. After washing, the membrane was developed with ECL system, and pictures were taken. The actin expression was used to normalize the protein expression levels using the ImageJ program.

### rVSVΔG-ZEBOV-GP infection in co-culture

Before the co-culture, HS primary cells were plated in a 24-well plate at a density of 3 × 10^5^ cells incubated for at least 24 h at 37°C with 5% CO_2_. Freshly isolated, CD14+ purified monocytes from healthy donors were plated in a separate 96-well round-bottom plate at a density of 3 × 10^5^/well, infected with rVSVΔG-ZEBOV-GP at different multiplicities of infection (MOIs 0, 1, 5, 10), and incubated for 1 h at 37°C with 5% CO_2_. For the co-culture, infected monocytes were extensively washed and plated onto the synoviocyte monolayer in the 24-well plates, resulting in 3 × 10⁵ monocytes and 3 × 10⁵ synoviocytes per well. All co-culture conditions were performed in 24-well plates with the same volume of fresh growth medium to ensure comparability across experiments. Supernatant was collected to quantify viral RNA by qRT-PCR at multiple time points post-infection.

### Luminex assay

Cryopreserved supernatants recovered after rVSVΔG-ZEBOV-GP *in vitro* infection were analyzed by Luminex (Magnetic Luminex assay, R&D Systems) to detect the concentration of 17 markers that form part of the reported innate plasma signature, previously shown in vaccinees to correlate with viremia, AEs, and hematological changes (13, 14). Assays were performed according to the supplier’s instructions. Briefly, beads conjugated with the biomarker-specific capture antibodies were incubated at RT for 2 h with samples, controls, or standards. Biotinylated detection antibodies and R-phycoerythrin–conjugated streptavidin were subsequently added. The mean fluorescence intensity of each marker was read on the Bio-Plex 200 array reader (Bio-Rad Laboratories) using the Luminex xMAP Technology (Luminex Corporation). A five-parameter logistic regression curve (Bio-Plex Manager 6.0) was used to calculate sample concentrations. Assessed cytokines and chemokines were IL-1Ra, MCP1, IL-6, IL-10, MIP1b, TNF-alpha, MCP2, MCP3, MCP4, CXCL10, CXCL11, CX3CL1, OSM, MCSF, TRAIL, RANKL, and IL15 (14).

### Flow cytometry

After *in vitro* infection with rVSVΔG-ZEBOV-GP of PBMCs, fluorescence-activated cell sorting (FACS) was performed to phenotype PBMC populations that were infected or not and to detect activation markers in gated monocyte populations. In the co-culture, FACS was used to detect infected synoviocytes. To assess the infection, cells were fixed and permeabilized (Thermo Fisher), and intracellular staining was performed using an anti-ZEBOV GP murine/human chimeric monoclonal antibody (c6D8) (IBT Bioservices).

After *in vitro* infection of healthy PBMCs with rVSVΔG-ZEBOV-GP, we performed phenotyping of PBMC populations. Cells were stained in PBS with LIVE/DEAD Fixable Aqua Dead Cell Stain Kit (Thermo Fisher) and FcR binding inhibitor (Miltenyi). Surface staining of the cells with a cocktail of anti-human antibodies allowed the identification of the different populations: monocytes (CD14+, CD16+/−, HLADR+, CD11C+, CD3−, CD20−, CD56−), T cells (CD3+, CD20−, CD14−, CD56−), NKT cells (CD3+, CD20−, CD14−, CD56+), B cells (CD3−, CD20+, CD14−), NK cells (CD14−, CD3−, CD20−, CD56+), pDCs (CD123+, CD11C−, CD3−, CD20−, CD56−), and mDCs (CD14−, CD16−, HLADR+, CD11C+, CD3−, CD20−, CD56−). In the same stain, the detection of activation markers was done on gated total monocyte populations (infected and non-infected), which were then gated for positive activation markers (CD86, CD169, CD163); gating was defined by using negative controls (non-infected PBMCs) and isotype controls.

For detection of synoviocytes in the co-culture, cells were stained in PBS with LIVE/DEAD Fixable Aqua Dead Cell Stain Kit (Thermo Fisher) and FcR binding inhibitor (Miltenyi). Surface staining of the cells with a cocktail of anti-human antibodies allowed the identification of synoviocytes such as CD14−, HLA-DR−, CD90+, and CD55+.

### Transcriptomic analysis of the co-culture

Quantification of gene expression in cell cultures was performed by RNA sequencing. The time point evaluated was 4 h after infection. Cells were then harvested by centrifugation at 1,500 rpm for 5 min and resuspended in lysis buffer RLT buffer with β-mercaptoethanol (Qiagen) for RNA extraction. Total RNA was extracted from human monocytes, synoviocytes, or co-culture using the Qiagen RNeasy Mini Kit, following the manufacturer’s protocol. Cells were lysed, homogenized, and RNA was purified using spin columns. Total RNA was quantified with Qubit (Thermo Fisher), and approximately 50 ng of total RNA was processed using Illumina Stranded mRNA Prep Ligation kit (Illumina) to prepare cDNA dual-indexed libraries according to the manufacturer’s recommendations. Dual-indexed libraries were purified using Agencourt AMPure XP Magnetic Beads (Beckman Coulter), quantified with NEBnext Library Quant Kit for Illumina (New England Biolabs) on a QuantStudio 5 Dx Real-time PCR System (Thermo Fisher), and their size was estimated on a BioAnalyzer DNA 1000 chip (Agilent Technologies, Germany). Libraries were diluted to a 0.5 nM concentration and pooled to a final volume of 100 µL for sequencing on a SP flow cell (Illumina), which was loaded according to the manufacturer’s protocol. Sequencing reactions were performed for 200 cycles on the Novaseq 6000 platform (Illumina) to obtain 100-base paired-end reads. Base-called data were quality-controlled using FASTQC, trimmed with TRIMMOMATIC, and then aligned either to the human reference genome (GRCh38.p7) using STAR (v2.4.2a) or to the rVSVΔG-ZEBOV-GP genome using BWA (v0.7.17). HTSEQ-COUNT tool was used to count the number of aligned reads for each gene.

### Data analysis

Graphs and statistics were generated using GraphPad Prism version 9.2.0. All data were log10 transformed and then two-way ANOVA analysis was performed, to correct for multiple comparisons we used Sidak’s test implemented in GraphPad Prism. *P* values ≤ 0.05are considered significant. We used the following designation of *P* values in graphs: ns, not significant; ****P ≤ 0.0001; ***P ≤ 0.001; **P ≤ 0.01; *P ≤ 0.05.

Differential gene expression analysis was performed using the R package DESeq2 (20) (v1.40.2). Since most genes did not show a normal distribution, differences in gene expression were assessed with the Wilcoxon test, corrected for multiple testing with the Benjamini-Hochberg method, on *vst* (variance stabilizing transformation) normalized data from DeSeq2. Enrichment analysis was performed with the clusterProfiler R package with the enrichKEGG and enrichGO functions. The background genes were those mapping to our data set. Statistical significance was assessed with the hypergeometric test, *P* values were adjusted using the Benjamini-Hochberg method to control the false discovery rate, and significance was set at an adjusted *P* value < 0.01.

## RESULTS

### In PBMCs, rVSVΔG-ZEBOV-GP infects mainly monocytes and induces their activation

**Fig 1:**
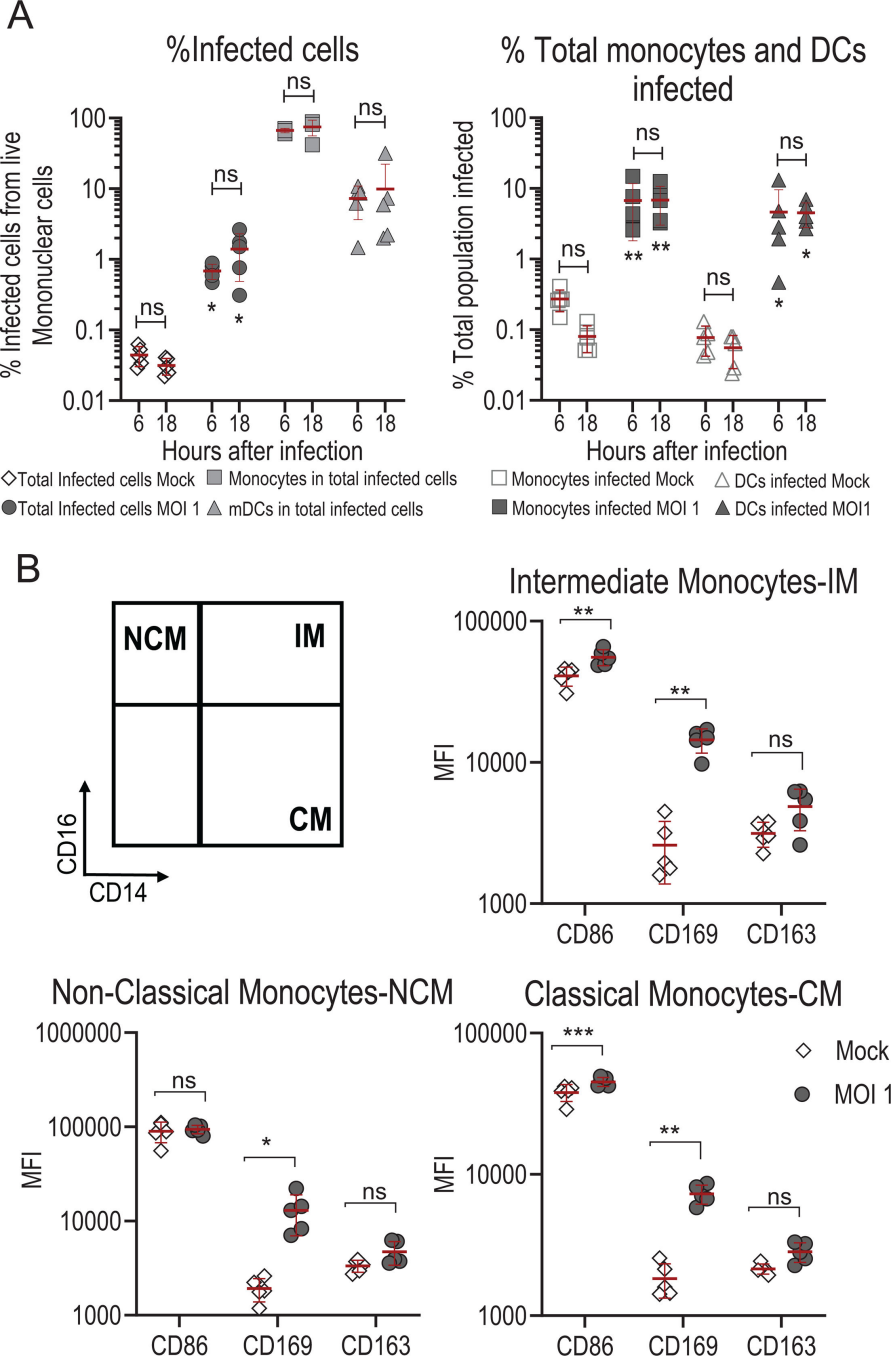
PBMC infection and activation markers after rVSVΔG-ZEBOV-GP *in vitro* inoculation. (**A**) Left: Percentage of infected cells at 6 h and 18 h after rVSVΔG-ZEBOV-GP *in vitro* inoculation. Total infected PBMCs (gray circle), with corresponding mock-infected controls (white circle), monocytes within total infected cells (white square), and myeloid dendritic cells (mDCs) within total infected cells (white triangle). Right: Percentage of total population infected at 6 h and 18 h after rVSVΔG-ZEBOV-GP *in vitro* inoculation, showing gated total infected monocytes after infection (square) and mock-infected controls (white square), gated total infected DCs after infection (gray triangle), and mock-infected controls (white triangle). (**B**) Geometric mean (GM) median fluorescence intensity (MFI) values for different activation markers expressed on total (infected and uninfected) monocyte populations (intermediate, classical, and non-classical) after rVSVΔG-ZEBOV-GP *in vitro* infection of PBMCs (gray circles) and mock-infected controls (white diamond). Each symbol represents a different donor (*n* = 5). MOI = 1 for all conditions. Two-way ANOVA analysis was performed. To correct for multiple comparisons, Sidak’s test implemented in GraphPad Prism was used. Asterisks indicate significant differences compared with mock-infected control: *P* < 0.05 (*), *P* < 0.01 (**), *P* < 0.001 (***); ns, not significant.

*In vitro* infection of PBMCs with rVSVΔG-ZEBOV-GP (detected as anti-VSV-NP-positive cells by flow cytometry) was used to assess the *in vitro* tropism of the vaccine to the different immune cell populations present in PBMCs (Fig. S1A and B). We tested two different time points for the *in vitro* infection of PBMCs and found that at 18 h there was no increase in percentage of total infected cells compared with 6 h (mean 0.69% [95% CI 0.49 to 0.89] and 1.39% [95% CI 0.27 to 2.51], *P* = 0.42) (Fig. 1A, left). We also showed that most of the infected cells are monocytes (overall mean 67% at 6 h and 75% at 18 h; *P* = 0.073), followed by myeloid dendritic cells (mDCs) (overall mean 7% at 6 h and 10% at 18 h; *P* > 0.999). Mock-infected controls were significantly lower than infected cultures (Fig. 1A left; Fig. S1D). Our data show no infection in other PBMC subsets (data not shown), meaning that the infection was largely restricted to monocytes and DCs. Additionally, we observed stable infection kinetics in PBMC cultures, where infected cells were detected by 6 h, and their levels remained unchanged at 18 h and 24 h (Fig. S1E), suggesting that most susceptible cells were infected early, with no further increase over time. When analyzing the total monocyte and DC populations, we observed that the percentage of infected cells was higher than in total PBMC: infected monocytes were on average 6.7% at 6 h and 6.8% at 18 h (*P* = 0.999), and infected DCs were on average 4.6% at 6 h and 4.5% at 18 h (*P* = 0.999) (Fig. 1A right; Fig. S1B and C). There was no significant difference in the percentage of monocytes and DCs infected cells between 6 h and 18 h, suggesting that the infection in these populations was well established by our first time point at 6 h.

Because monocytes and mDCs are the main subsets infected, we then analyzed the expression of activation markers in these subsets 18 h after *in vitro* infection and used the non-infected mock condition to evaluate whether the expression was induced by the infection. All monocyte populations (classical monocytes [CM], intermediate monocytes [IM], and non-classical monocytes [NCM]) showed significant increase in the median fluorescence intensity (MFI) for CD169 after infection compared to non-infection. Only CM and IM showed a significant increase in the MFI for CD86 after infection compared to non-infection, while CD163 MFI was not statistically significant in any monocyte subset (Fig. 1B). We also compared infected monocytes to non-infected monocytes within the same culture and found no significant difference in activation marker expression between the monocyte subsets (data not shown).

These results confirmed that, in PBMCs, rVSVΔG-ZEBOV-GP has a tropism for monocytes and mDCs and that activation of the different monocyte populations is not strictly dependent on direct infection but rather a result of exposure to the virus or the infected environment.

### CD14+ monocytes produce plasma innate signature markers after *in vitro* rVSVΔG-ZEBOV-GP infection

**Fig 2:**
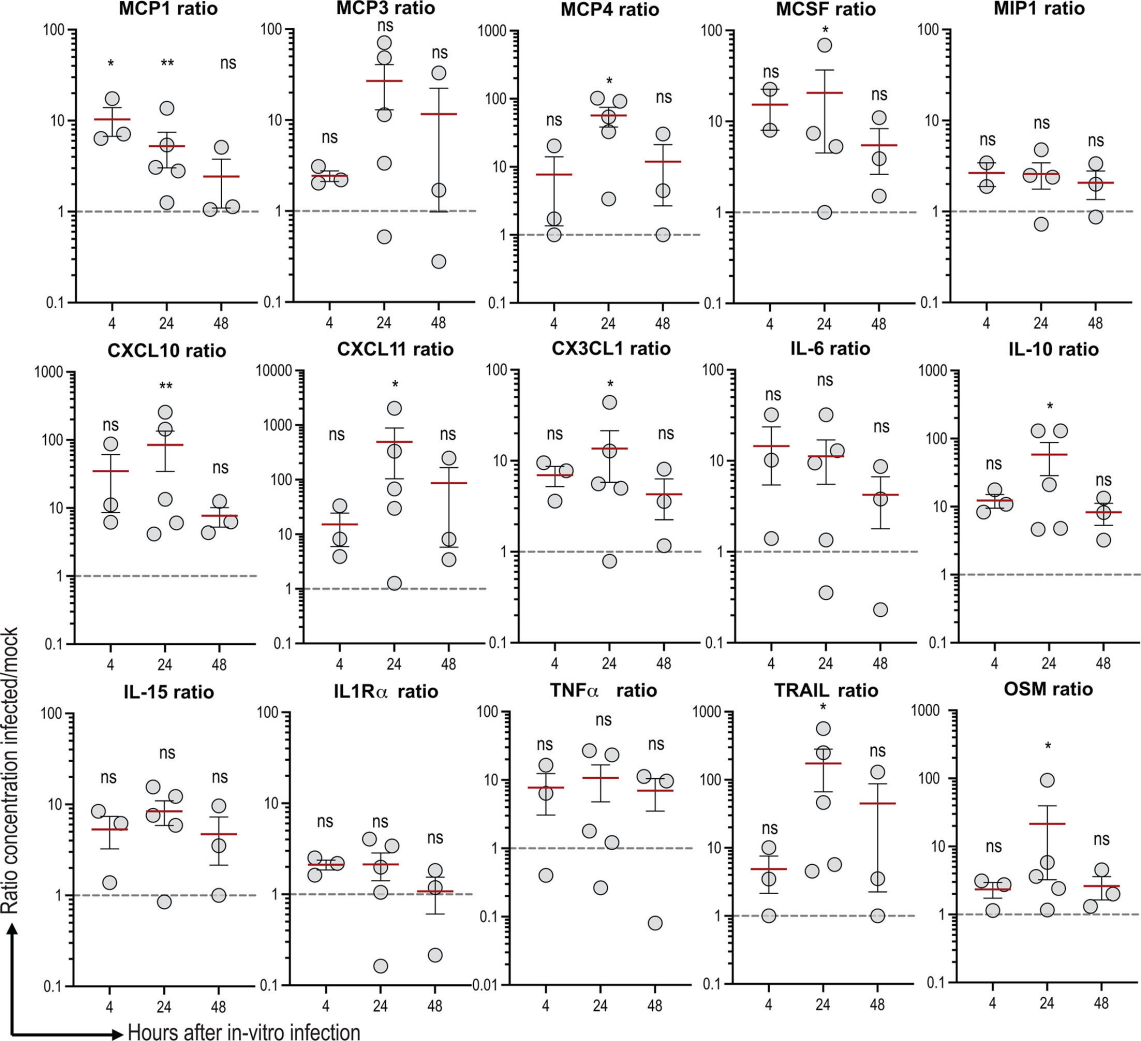
Pure monocyte cytokine production after rVSVΔG-ZEBOV-GP *in vitro* inoculation. For visualization, cytokine secretion in supernatants is shown as infected/mock ratios (mock = 1). Each circle represents a donor, the red line represents the mean, and the error bar represents SEM (*n* = 3: 6 h and 48 h; *n* = 5: 24 h). For statistical analysis, paired raw values (infected vs mock) were compared using two-way ANOVA with Sidak’s correction for multiple comparisons. Asterisk represents *P*-value: *P* < 0.05 (*), *P* < 0.01 (**); ns, not significant.

We evaluated whether isolated CD14+ monocytes were producers of innate plasma signature biomarkers (i.e., 17 cytokines and chemokines) reported recently (14). After rVSVΔG-ZEBOV-GP *in vitro* infection of CD14+ monocytes, all plasma signature markers were detected in culture supernatant as soon as 6 h post-infection, reached a peak at 24 h, and were still detectable at 48 h. The background level in non-infected CD14+ monocytes was high, and the levels were very heterogeneous; therefore, some markers were significantly higher only at 24 h compared to baseline: MCP-1, MCP-4, MCSF, CXCL10, CXCL11, CX3CL1, IL-10, OSM, and TRAIL (Fig. 2). These results demonstrate that isolated CD14+ monocytes infected with rVSVΔG-ZEBOV-GP can produce biomarkers previously identified in the innate plasma signature, with significant increases in several cytokines at 24 h post-infection compared to baseline.

### Cellular tropism of rVSVΔG-ZEBOV-GP vaccine virus in primary human cells and human cell lines

We next assessed the *in vitro* cellular tropism of rVSVΔG-ZEBOV-GP in human primary cells from tissues previously associated with unexpected AEs, such as synoviocytes, fibroblasts, keratinocytes, endothelial cells, and chondrocytes.

**Fig 3:**
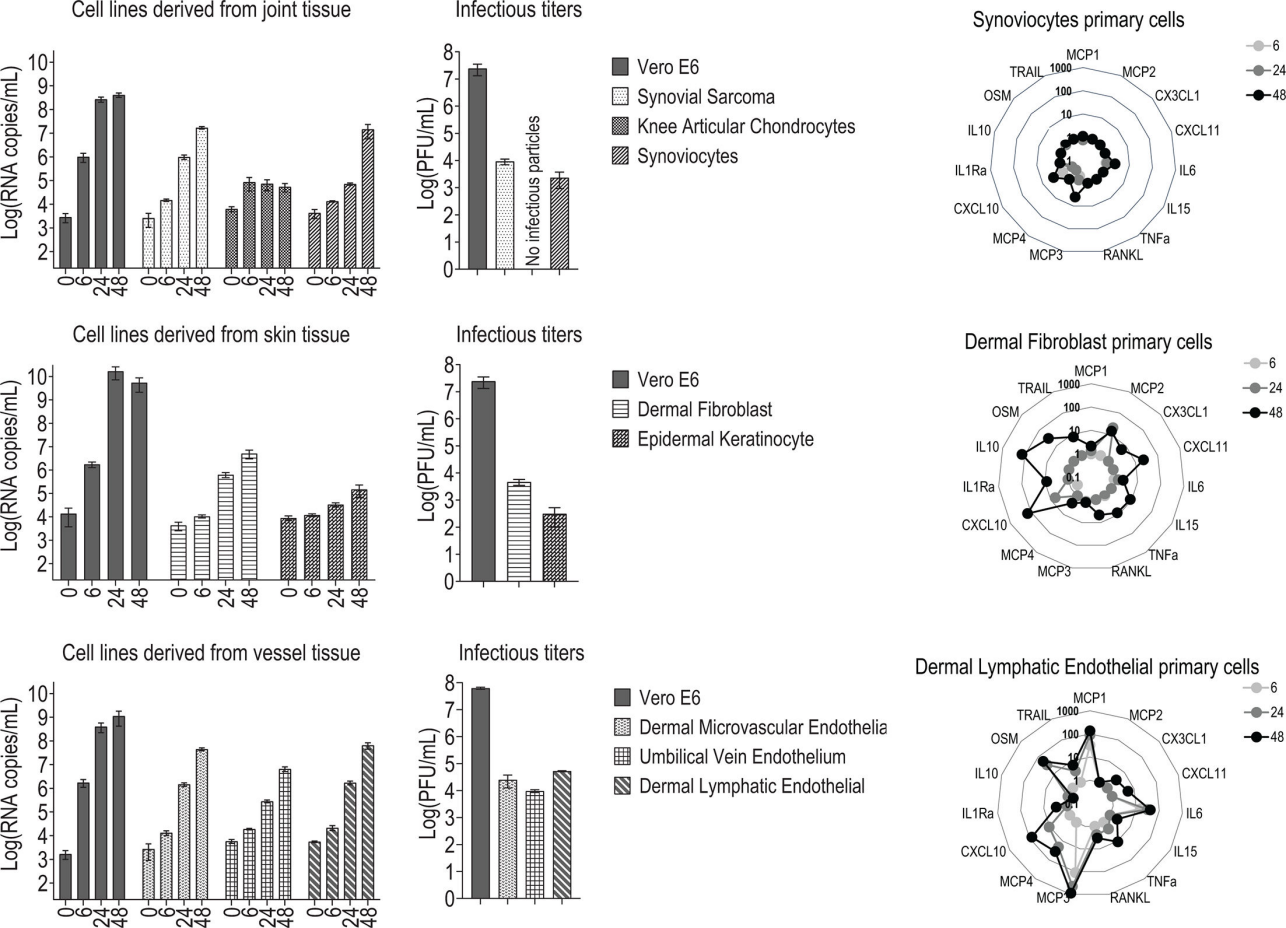
Viral replication, plaque assay, and cytokine response in primary cells derived from joint, skin, and vessels compartments. Viral loads were measured in supernatant by RT-qPCR at 0 h, 6 h, 24 h, and 48 h after *in vitro* rVSVΔG-ZEBOV-GP infection (MOI 0.1) and are expressed as mean ± SEM (*n* = 4). The 0 h samples were included as baseline controls to capture residual input virus. Infectious titers were calculated by performing a plaque assay in Vero cells at 24 h after *in vitro* infection. Viral replication and plaque assays are shown as bar graphs. Cytokine responses were analyzed at 6 h, 24 h, and 48 h by Luminex assay, shown as a ratio of MOI 0.1 versus mock, and are expressed as mean ± SEM (*n* = 2) of two biological replicates (with technical duplicates) and are presented descriptively as spider plots.

In tissue-derived primary joint cell lines, *in vitro* viral replication increased rapidly over time, with an increase of almost 4 log_10_ RNA copies/mL 48 h after infection in human synovial sarcoma (cell line) and synoviocytes (primary cells) (Fig. 3, left). In skin tissue primary cells, we observed *in vitro* viral replication in both human dermal fibroblasts and human epidermal keratinocytes, which was higher in fibroblasts (Fig. 3, left). All human endothelial primary cells tested (dermal microvascular, umbilical vein, and dermal lymphatic endothelial cells) showed an increase of 4 log_10_ RNA copies/mL after *in vitro* infection (Fig. 3, left). T0 samples were included as baseline controls to capture residual input virus. The plaque assay confirmed that infectious particles were formed after *in vitro* infection in all these primary cells, except human knee articular chondrocytes, in which neither viral replication nor infectious viral particle production was observed (Fig. 3 middle).

In the supernatant, we observed secretion of innate plasma signature markers after rVSVΔG-ZEBOV-GP *in vitro* infection. A tendency to increase over time was detected mainly in lymphatic endothelial cells (MCP3, MCP4, MCP1, IL-6, CXCL10, OSM, TRAIL, and TNF-alpha), followed by skin fibroblast (CXCL10, CXCL11, CX3CL1, MCP2, IL10, IL15, TNF-alpha, RANKL, and OSM), while synoviocytes only secreted CXCL10 and MCP3 in a lower amount (Fig. 3 right; Fig. S2). Cytokine concentrations from mock-infected controls are shown in Fig. S2. Microvascular and vein endothelial cells produced mainly CXCL10 48 h after *in vitro* infection (Fig. S3A). We also found that chondrocytes did not produce these markers over time, all arguing against viral replication in these cells (Fig. 3 right; Fig. S3A).

Next, we determined whether the permissiveness of these primary cells is associated with the presence of EBOV receptors T-cell immunoglobulin and mucin domain 1 (TIM-1) and Niemann-Pick C1 (NPC1). NPC1 protein was present in all primary cell lines tested, while TIM-1 detection was low or absent in some. However, in the non-infectable chondrocytes, both NPC1 and TIM-1 were clearly detected, suggesting that the presence of these receptors alone may not be sufficient to confer susceptibility to infection (Fig. S3B).

We showed that several cell lines of the tissue previously associated with AEs, such as synoviocytes, fibroblasts, keratinocytes, and endothelial cells, were productively infected *in vitro* by rVSVΔG-ZEBOV-GP. The *in vitro* infection induced the secretion of several innate plasma signature biomarkers, especially in dermal fibroblasts and dermal lymphatic endothelial cells, compared to mock-infected controls.

### Infected monocytes transmit infection into synoviocytes and modulate innate response

**Fig 4:**
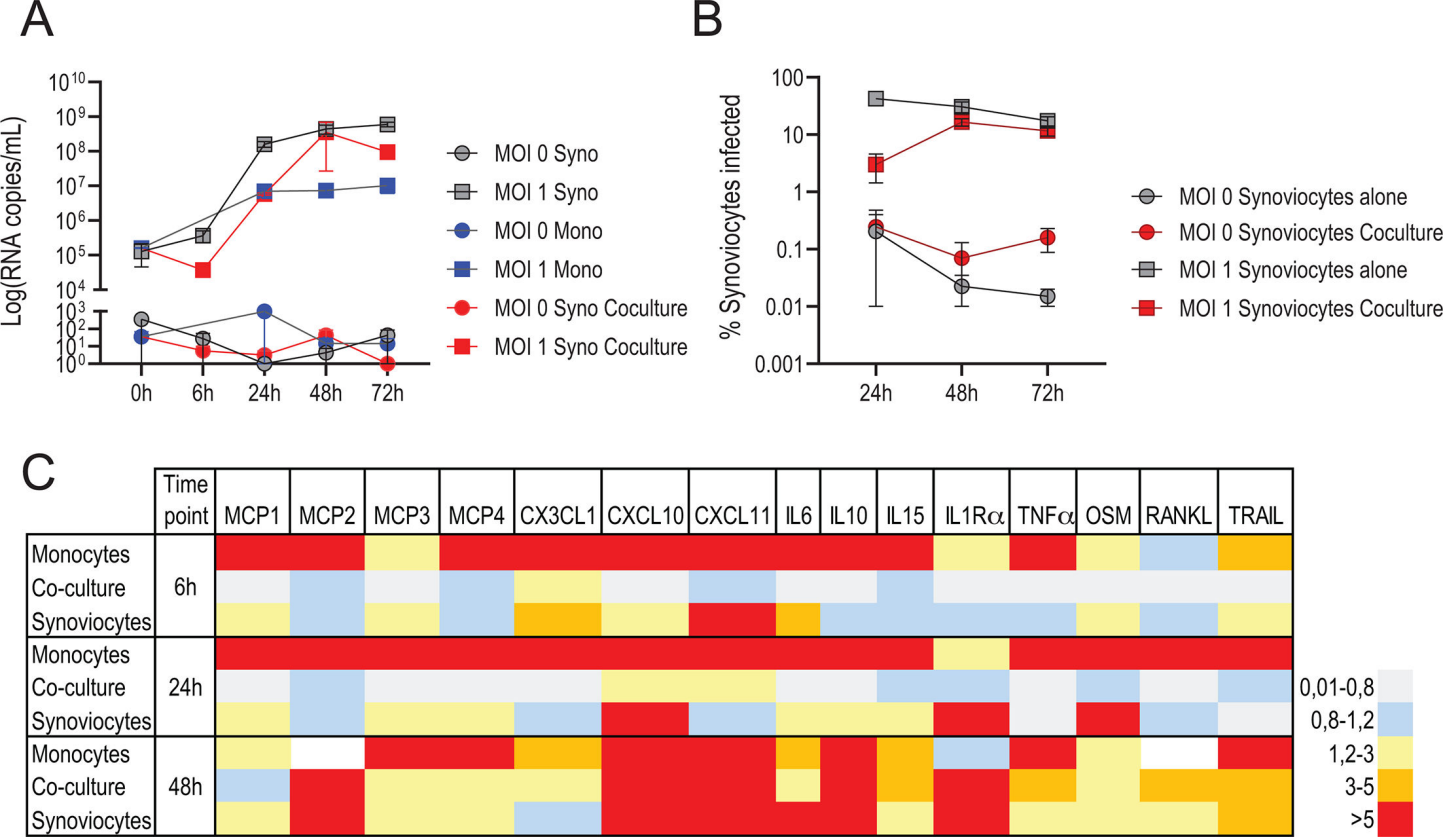
Co-culture viral replication and cytokine response. (**A**) Viral loads (left) were measured in supernatant by RT-qPCR at 0 h, 6 h, 24 h, 48 h, and 72 h after *in vitro* rVSVΔG-ZEBOV-GP infection (MOIs 0 and 1) of synoviocytes (black), monocytes (blue), or co-culture (red) and are expressed as mean ± SEM (*n* = 2). (**B**) Percentage of infected synoviocytes after *in vitro* rVSVΔG-ZEBOV-GP infection (MOIs 0 and 1) of synoviocytes alone (black) or synoviocytes in co-culture with monocytes (red). MOI 0 (circle). MOI 1 (square). (**C**) Heat map shows the mean (*n* = 3) of the ratio (MOI 1 over mock control) for each marker except RANKL (*n* = 1) at different time points (6 h, 24 h, and 48 h) in different populations after *in vitro* rVSVΔG-ZEBOV-GP infection (MOI 1). Markers with values higher at mock are in blue, markers with similar value between MOI and mock are in light gray, and markers with values higher at MOI than in mock are shown from (low) yellow to red (high).

Then, we evaluated whether infected monocytes (the main blood population permissive to rVSVΔG-ZEBOV-GP infection) could infect synoviocytes (Fig. S4A). In the co-culture, the kinetics of viral replication differed from that observed in monocytes and synoviocytes. Specifically, replication appeared slower compared to synoviocyte infection alone but appeared faster compared to monocyte infection alone. Viral RNA was first detected 6 h post-infection and peaked at 48 h (mean 3.56 × 10^6^ copies/mL of supernatant). This replication pattern was not influenced by the viral dose, as similar results were observed at MOI 1 (Fig. 4A), MOI 5, and MOI 10 (Fig. S4B). To identify the percentage of infected synoviocytes in the co-culture, synoviocytes were defined by FACS using the morphological gate as live cells and the markers CD14−, HLA-DR−, CD90+, and CD55+ (Fig. S4C), and infected synoviocytes were defined as the gated synoviocytes that were VSV-NP+ (Fig. S4D). In the co-culture, we observed that synoviocytes were infected, although with a slower kinetics compared to synoviocytes-alone culture at 24 h (mean 2.9%, SD 2.2) (Fig. 4B). By 48 h, infected synoviocytes in the co-culture reached similar levels as synoviocytes-alone culture (mean 16.5%, SD 3.5), which were maintained at 72 h (Fig. 4B) (mean 11.61%, SD 3.1). Similar results were observed when the viral inoculum was increased (MOIs 5 and 10) (Fig. S4D).

We also evaluated whether the interaction of the monocytes and the synoviocytes in the co-culture had an impact on the secretion of the innate signature markers. In the co-culture, we observed that the secretion kinetics of the innate signature markers were intermediate: appeared slower compared to monocytes alone and appeared faster compared to the synoviocytes alone at both 6 h and 24 h post-infection, and it was reestablished to similar levels as to the single culture cells at 48 h for most of the markers except MCP1 (Fig. 4B).

These results suggest that *in vitro* rVSVΔG-ZEBOV-GP-infected monocytes produced infectious particles, which served as the source of synoviocyte infection in co-culture, with an associated modulation of biomarker secretion kinetics. However, because co-cultures and monocyte-only conditions were performed in different well formats, this apparent delay should be interpreted with caution.

### Transcriptomic analysis indicates an inflammatory response to rVSVΔG-ZEBOV-GP infection

We then analyzed the transcriptomic profile of monocytes, synoviocytes, and their co-cultures in response to 4 h *in vitro* infection with rVSVΔG-ZEBOV-GP. Viral gene expression was about 100-fold and 2-fold higher in the synoviocytes and monocytes, respectively, compared to the co-culture (Fig. S5). Differential gene expression analysis identified relatively few significant (FDR < 0.05) differentially expressed (DE) genes in infected monocytes and co-cultures compared to the uninfected cells, while more than 10% of the expressed genes were DE in infected synoviocytes (Table S2). Gene enrichment analysis showed that there was a conserved response to infection involving modules related to innate immunity activation, including RIG-I signaling, activation of dendritic cells, interferon response, and chemokines (Figure S6). Infected synoviocytes were enriched in modules related to the regulation of cell proliferation and death. While in infected monocytes, transcriptomic analysis revealed no induction of IFNG, IL1B, or TNF, whereas TLR3 expression was increased compared to mock-infected controls, while TLR4, TLR7, and TLR8 remained unchanged (Fig. S7), which indicates activation of antiviral sensing pathways without a concomitant pro-inflammatory cytokine response.

**Fig 5:**
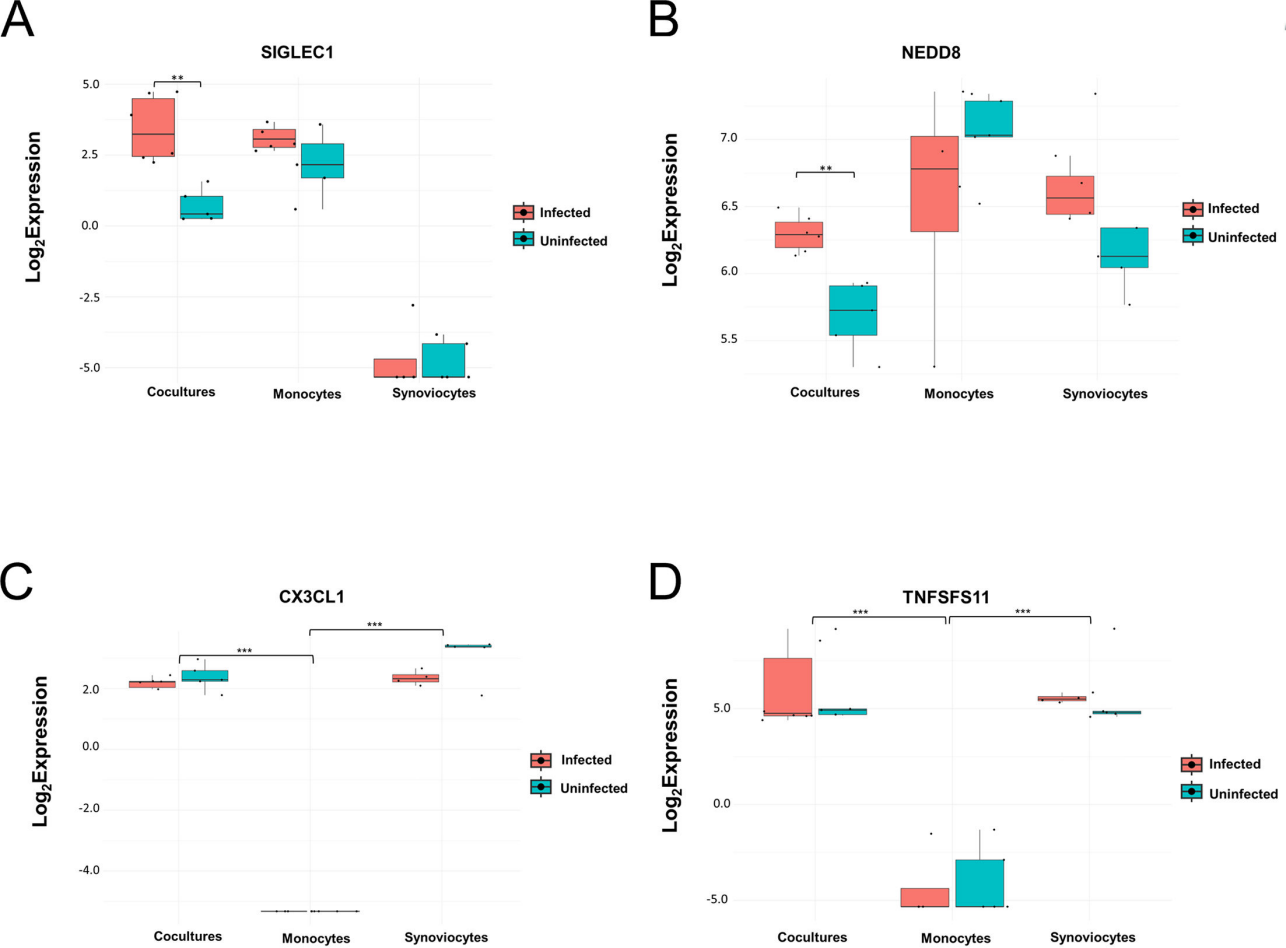
Normalized expression (log_2_ expression) levels of selected genes in different cell cultures. (**A**) SIGLEC1, (**B**) NEDD8, (**C**) CX3CL1, and (**D**) TNFSFS11 expression levels in infected and uninfected cell cultures. Data are reported as box and whiskers plot, where the marked line inside the box represented the median value, the box the interquartile range (IQR), and whiskers the minimum and maximum values in the range ±1.5 × IQR. Individual values are reported as black dots. Differences in gene expression between infected and uninfected samples (**A and B**) or among different cell culture types (**C and D**) were assessed with the Wilcoxon test (**P < 0.01, ***P < 0.001).

A comparison of DE genes in the different cell lines identified 12 genes that were unique to the co-culture (Fig. S8). Among these genes, it is worth mentioning *NEDD8*, which encodes a ubiquitin-like protein possibly involved in the inflammatory arthritis pathogenesis (21), and *SIGLEC-1*, which encodes the CD169 macrophage surface marker (Fig. 5A and B). Both genes are only upregulated in the infected co-culture, indicating that the interaction of infected monocytes with synoviocytes triggered their activation. Coherently with the results of the Luminex assay, the expression levels of cytokine/chemokine genes were generally lower in the co-culture compared to monocytes (Fig. S9), except for *TNFSF11*, which codes for RANKL, *CX3CL1*, and *IL6*, which were more abundant in the synoviocytes and in the co-culture under both infected and uninfected conditions (Fig. 5C and D). Interestingly, the genes coding for EBOV receptors, *NPC-1* and *TIM-1,* were more expressed in the co-culture compared to both monocytes and synoviocytes, and their expression did not increase upon infection with rVSVΔG-ZEBOV-GP (data not shown).

## DISCUSSION

In this study, we investigate the complex events that unfold after infection by studying *in vitro* the cellular tropism of the rVSVΔG-ZEBOV-GP vaccine. Our findings revealed that in cell culture, endothelial cells, fibroblasts, keratinocytes, synoviocytes, and peripheral monocytes supported the infection and production of infectious viral particles. Among these permissive cells, peripheral monocytes were found to secrete most innate signature biomarkers, indicating their significant role in the early immune response to the vaccine viral infection. Upon rVSVΔG-ZEBOV-GP infection, monocytes got activated, and when co-cultured with synoviocytes, were able to transmit the infection to synoviocytes *in vitro*. Furthermore, the interaction between monocytes and synoviocytes appeared to modulate the kinetics of innate response signature biomarkers secretion and upregulation of 12 genes, including *NEDD8* and *SIGLEC-1*.

We evaluated *in vitro* the tropism of rVSVΔG-ZEBOV-GP in PBMCs and showed that monocytes and dendritic cells were the only permissive cells for rVSVΔG-ZEBOV-GP infection in PBMCs. These results confirm that the vaccine’s tropism in PBMCs is limited to these two populations, and as expected, it mirrors the tropism of the EBOV glycoprotein (GP) (22). Even if NK cells are modulated after rVSVΔG-ZEBOV-GP vaccination (23), they were not permissive for the infection. This is consistent with previous *in vitro* studies, which have shown that the EBOV GP is a ligand for TLR-4 and induces activation of uninfected monocytic cell lines, monocyte-derived DCs, and macrophages to produce cytokines (24, 25).

We also showed that infection with rVSVΔG-ZEBOV-GP induced activation of both CM and IM, but neither of mDCs nor NCM, similar to what has been observed after vaccination in humans, where CD86 was significantly increased already at one day after vaccination in monocytes (26). CD169 was upregulated in all three monocyte subsets upon infection, suggesting possible differentiation of monocytes into macrophages. This is consistent with the transcriptional upregulation (27, 28) and increased surface expression (5) of CD169 observed after rVSVΔG-ZEBOV-GP vaccination in humans, which is also consistent with the critical role of CD169^+^ macrophages to induce protective innate and adaptive responses following rVSVΔG-ZEBOV-GP vaccination in mice (29). In contrast to what has been shown for SARS-CoV-2 infection, where monocytes abort the replication and infected particles are not detected (30), here we show that monocytes allowed the replication of the rVSVΔG-ZEBOV-GP and also produce infectious viral particles, reinforcing their role in the vaccine innate response (13, 14). Upon rVSVΔG-ZEBOV-GP *in vitro* infection, monocytes produced only a subset of innate plasma signature biomarkers (MCP-1, MCP-4, M-CSF, CXCL10, CXCL11, CX3CL1, IL-10, OSM, TRAIL), whereas in the plasma of vaccinees, all markers tested were increased (14), indicating that additional cell types contribute to the vaccine-induced signature. As transcriptomic studies of rVSVΔG-ZEBOV-GP vaccination have shown early induction of interferon-stimulated genes (ISGs) (27), we assessed these pathways in our *in vitro* monocyte model. We observed upregulation of TLR3, consistent with viral RNA sensing, but no induction of IFNG, IL1B, or TNF, indicating a limited pro-inflammatory response.

We observed production of many innate signature biomarkers after rVSVΔG-ZEBOV-GP *in vitro* infection of dermal lymphatic endothelial cells and dermal fibroblasts, mainly monocyte chemotactic proteins such as MCP1, MCP3, and MCP4 as well as CXCL10, TRAIL, TNF-α, and OSM. Interestingly, TRAIL has been shown to promote apoptosis of keratinocytes (31), and OSM has been implicated in driving inflammation in mouse models where it was highly overexpressed (32). Moreover, TNF-α was secreted by both dermal fibroblasts and lymphatic endothelial cells, potentially amplifying local inflammatory signaling (33). Given that unexpected AEs, such as dermatitis and cutaneous vasculitis, reported after rVSVΔG-ZEBOV-GP vaccination were not always linked with the presence of the virus vaccine in the skin biopsy (5), we suggested that dermatitis and cutaneous vasculitis might be the result of indirect effects of these secreted cytokines, as has been shown in EBOV disease-related disruption of the vascular endothelium (34).

Interestingly, our findings show that primary human chondrocytes were not permissive to rVSVΔG-ZEBOV-GP infection, despite expressing the known EBOV entry receptors TIM-1 and NPC1 (35, 36). This suggests that receptor presence alone does not guarantee permissiveness to infection and indicates the involvement of additional post-entry restrictions. Similar observations have been reported in other cell types, where intracellular antiviral mechanisms override receptor-mediated susceptibility (37, 38). One plausible explanation is the activation of potent intrinsic or innate immune responses in chondrocytes. These cells may constitutively express or rapidly induce antiviral effectors, including type I or III interferons and ISGs that inhibit viral replication at early stages (39). In particular, restriction factors, such as IFITM proteins, which have been shown to block endosomal fusion events of various enveloped viruses, could contribute to the observed resistance (40–42). Moreover, a lack of host factors required for glycoprotein priming or fusion, such as specific cathepsins, may further limit productive infection in this cell type (43). However, the precise mechanisms underlying this resistance remain unclear and warrant further investigation. Future studies should focus on characterizing the baseline and infection-induced expression of innate immune mediators and restriction factors in chondrocytes. Understanding these cell type-specific antiviral barriers may provide important insights into rVSV-ZEBOV vaccine safety and the differential susceptibility of human tissues to viral vectors.

We showed that synoviocyte cell lines *in vitro* infection with rVSVΔG-ZEBOV-GP supported viral replication and produced infectious virus, as indicated by increasing viral RNA levels and the detection of infectious particles in supernatants by plaque assay. This is in line with the capacity of EBOV to infect monkey synoviocytes *in vivo* and *in vitro* (44). While viral RNA was consistently detected in infected synoviocytes, the limited release of infectious particles in plaque assays suggests a predominantly transient infection. The presence of viral RNA is sufficient to trigger innate immune responses through cytoplasmic RNA sensors, such as RIG-I and MDA5, which are expressed in synoviocytes (45, 46). Notably, we observed that synoviocyte secretion of innate signature markers was dose-dependent on the viral inoculum, supporting the link between viral exposure and local inflammation. Although arthritis was reported in patients who survived EBOV disease (47), the transient arthritis reported after rVSVΔG-ZEBOV-GP vaccination was an unexpected AE (5, 12). Previously, we showed that transient arthritis after rVSVΔG-ZEBOV-GP vaccination develops in individuals with a lower level of innate inflammatory plasma signature response after rVSVΔG-ZEBOV-GP vaccination, and we believe that these individuals have less effective early control of viral dissemination (14), which may in turn lead to viral presence in privileged sites such as joints. In the co-culture, our data suggest that rVSVΔG-ZEBOV-GP-infected monocytes may be a source of infected particles that subsequently infected synoviocytes, and we also observed a modulation in the secretion of the innate signature markers. Altogether, it suggests that the infected monocytes can act as a Trojan horse to allow the virus to reach immune-privileged sites, such as the joints, which, together with the modulation of the innate response, could propitiate an environment for viral replication and transient viral arthritis. In the co-culture, we also observed the reduction of bone resorption markers, such as RANKL (secretion level), as well as molecules that promote osteoclast differentiation, such as TRAIL (at both transcriptional and secretion levels) (48). This is in line with the absence of bone resorption lesions in transient arthritis after rVSVΔG-ZEBOV-GP vaccination (5), in contrast to chikungunya arthritis (49). However, the observed upregulation of *NEDD8* in the co-culture and infected synoviocytes points to a transcriptional program involved in post-transcriptional modifications associated with the inflammatory processes in arthritis (21) and fibroblast-like synoviocyte inflammation (50). Similarly, SIGLEC-1 has been implicated in inflammatory responses and synovial activation (51). Importantly, both NEDD8 and SIGLEC-1 also play roles in protective immune responses to vaccination, reflecting their context-dependent functions (52, 53).

Our study had some limitations. The number of samples was low, ranging from 2 to 5, mainly due to the complexity of the experiments, which involved measuring different time points, different viral inoculum, and different readouts. Monocytes are well known to be easily activated *in vitro* upon contact with plastic materials. To minimize this activation, we used round-bottom well plates made of low-adherence treated plastic. Although we observed a clear increase in monocyte activation markers after infection through FACS, the uninfected condition showed a high basal background, which was also present in the supernatant quantification of innate markers. We also used different MOIs for the infection of PBMCs/monocytes and primary cells because our readout for monocyte infection was through FACS, and an MOI of less than 1 required longer incubation times, increasing the risk of non-specific activation. It is important to note that our study was conducted in an artificial *in vitro* system using an elevated infectious dose (MOI 1 for PBMCs and MOI 0.1 for primary cells) to maximize infection efficiency and assess off-target cell susceptibility. In addition, our co-culture model was simplified, as it did not include other immune populations, such as T cells, neutrophils, or additional synovial-resident cells that may influence inflammatory kinetics *in vivo*. While this approach allowed us to characterize *in vitro* cellular tropism and innate immune responses, it also means that caution is needed when extrapolating our findings to *in vivo* conditions. Further studies in humans or in more complex models, such as organoids, will be valuable to assess the relevance of these observations.

In conclusion, we identified *in vitro* off-target cellular tropism of rVSVΔG-ZEBOV-GP, infecting several cell types from tissues previously associated with unexpected AEs, such as synoviocytes, fibroblast, keratinocytes, and endothelial cells. These cells were productively infected by rVSVΔG-ZEBOV-GP and secrete specific innate pro-inflammatory biomarkers. Monocytes were the main population supporting rVSVΔG-ZEBOV-GP *in vitro* infection in PBMCs, became activated, and secreted most innate plasma signature markers upon *in vitro* infection and may potentially serve as a source of synoviocyte infection *in vitro*. Additionally, co-culture revealed that interactions between infected monocytes and uninfected synoviocytes modulate the innate immune response, altering the secretion kinetics of biomarkers; however, this should be interpreted with caution given differences in experimental setups between single- and co-cultures.

Altogether, our study provides an example of how *in vitro* models can be used to study off-target vaccine virus tropism and the interplay between immune cells and primary cells. However, further studies are needed to determine *in vivo* relevance of these off-target infections, their role in early and sustained immune responses to the vaccine.

## Data Availability

The RNA-seq data generated in this study have been deposited in the NCBI Gene Expression Omnibus (GEO) under the accession number GSE296388. All raw and processed files, including sample metadata and normalized expression matrices, are included in the submission.

## SUPPLEMENTAL MATERIAL

Supplemental material spectrum.00408-25-s0001.pdf Fig. S1 to S9; Tables S1 and S2.

ASM does not own the copyrights to Supplemental Material that may be linked to, or accessed through, an article. The authors have granted ASM a non-exclusive, world-wide license to publish the Supplemental Material files. Please contact the corresponding author directly for reuse.
