# Supplementary material for: In vitro cellular tropism and immunomodulatory response to rVSVΔG-ZEBOV-GP in human cells derived from tissues associated with adverse events
Source: Microbiol Spectr. 2026 Mar 24;14(5):e00408-25. doi: 10.1128/spectrum.00408-25 (PMC13141887; doi:10.1128/spectrum.00408-25)
Supplement: Supplemental material — Fig. S1 to S9; Tables S1 and S2. [file spectrum.00408-25-s0001.pdf]

Supplementary figure 1

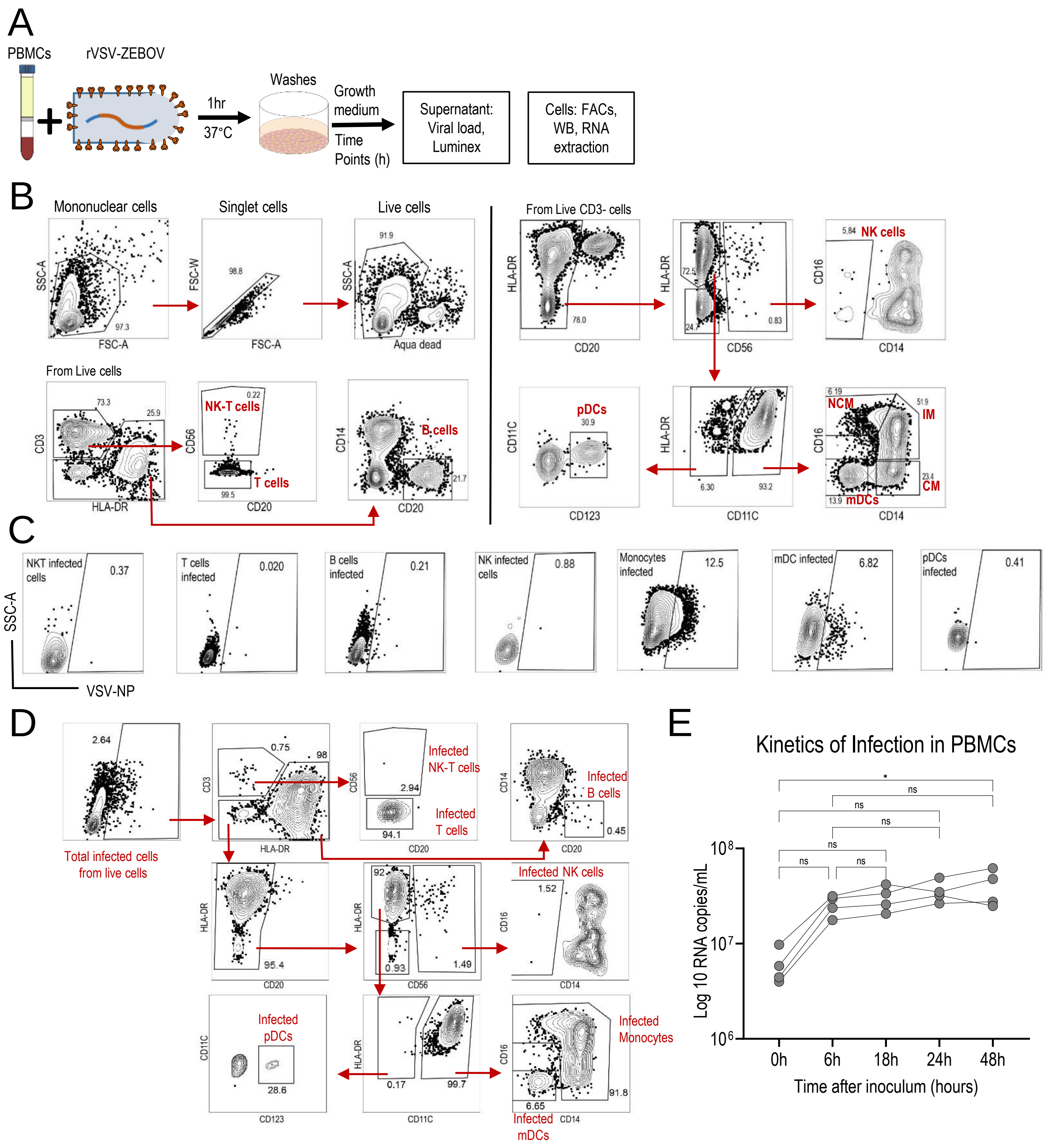

Supplementary figure 1: PBMCs in-vitro infection and FACS of populations evaluated. (A). Scheme of the in-vitro infection of PBMCs. **(B)** Gate strategy for all populations evaluated. NK cells (live cells, CD3-, CD20-, CD14-, CD56+, CD16+/-); NK-T cells like (live cells, CD20-, HLA-DR-, CD3+, CD56+); T cells (live cells, CD20-, HLA-DR-, CD3+, CD56-); B cells (live cells, CD3-, CD14-, CD20+); Classical Monocytes-CM (live cells, CD3-, CD20-, HLA-DR+, CD11C+, CD14+, CD16-); Non-Classical Monocytes-NCM (live cells, CD3-, CD20-, HLA-DR+, CD11C+, CD14+, CD16-); Intermediate Monocytes-IM (live cells, CD3-, CD20-, HLA-DR+, CD11C+, CD14+, CD16+). **(C)** FACS plots showing populations infected (VSV-NP+) from a representative sample at 18h after in-vitro infection (MOI 1). (D) FACs gating strategy showing infected populations from total infected cells. (E). Kinetics of infection efficiency in PBMC cultures at MOI 1, assessed by measuring viral RNA in the supernatant using RT-qPCR (n=4).

Supplementary figure 2

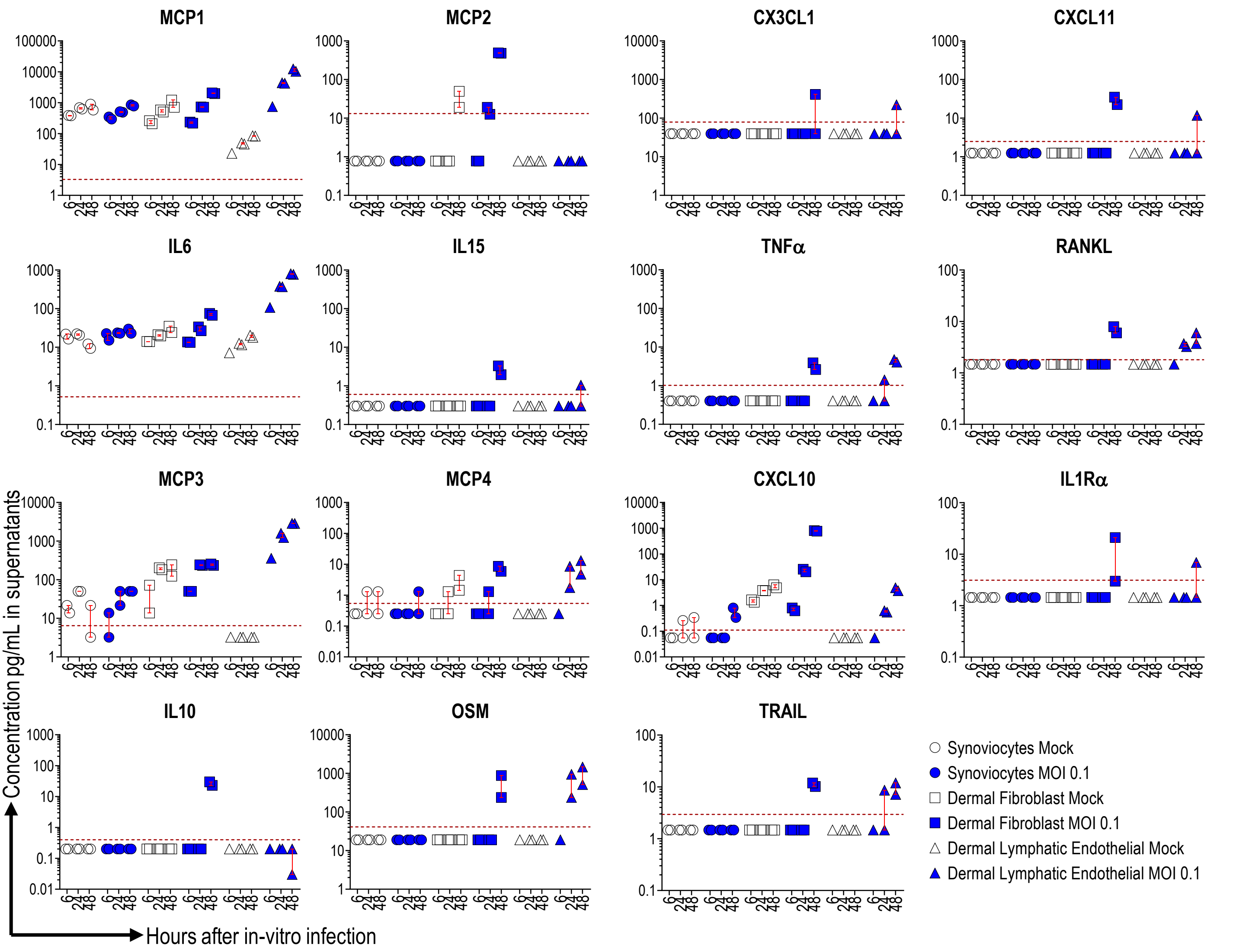

Supplementary Figure 2: Cytokine response after in-vitro rVSVΔG-ZEBOV-GP infection. Luminex concentration in supernatants (pg/ml) for each marker was plotted at each time point (6h, 24h, 48h) after rVSVΔG-ZEBOV-GP in-vitro inoculation in the different groups: mock (white diamond) and MOI 1 (pink circle). Results are expressed as mean ± SEM (n=2).

Supplementary figure 3

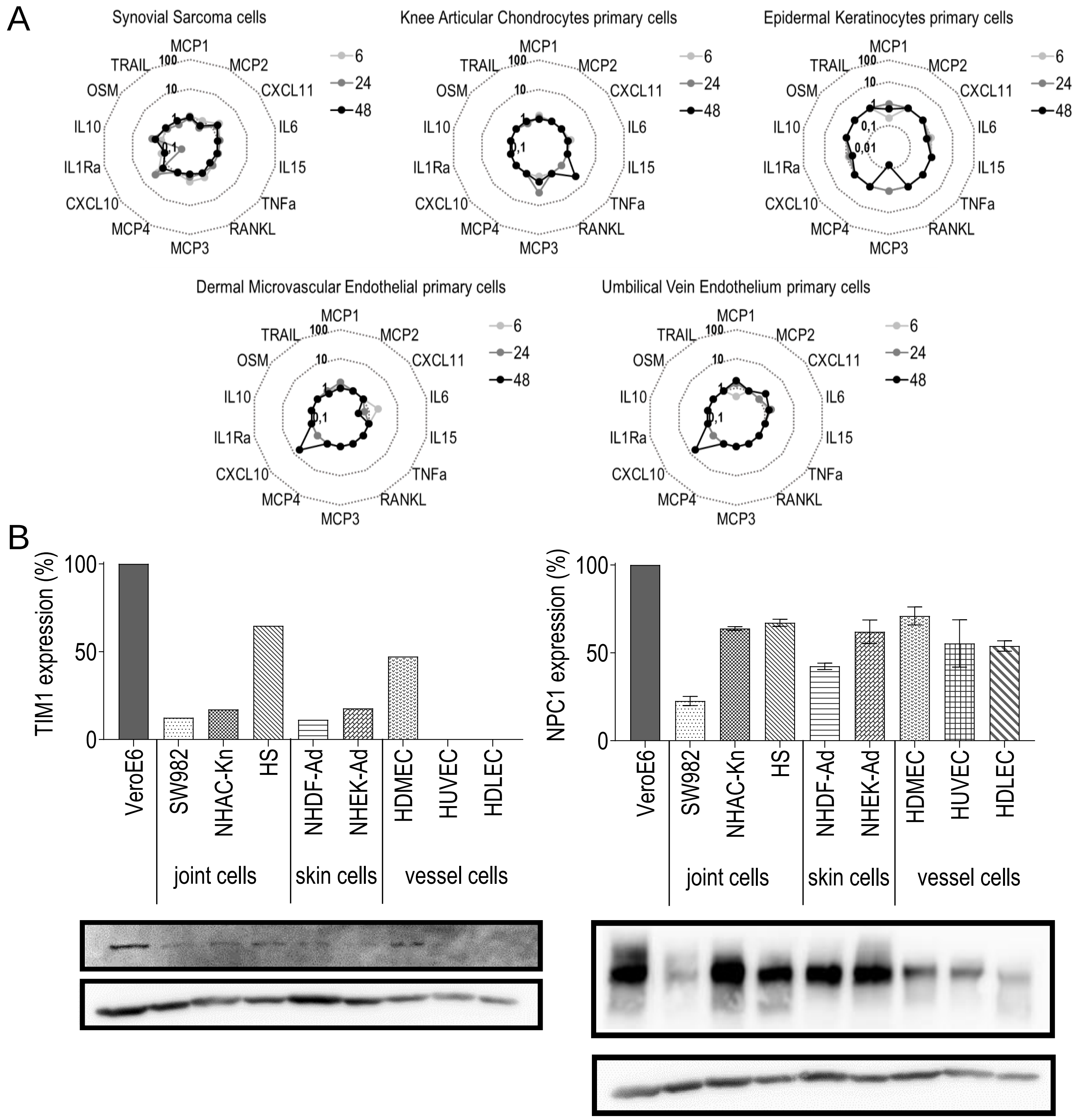

Supplementary Figure 3: Cytokine response after in vitro rVSVΔG-ZEBOV-GP infection and Ebola receptors expression. (A) The cytokine response was analysed at 6hrs, 24hrs and 48hrs after in vitro rVSV-ZEBOV infection by Luminex assay by doing a ratio between infection at MOI 0.1 versus Mock in joint, skin and vessel cells and are expressed as mean ± SEM (n=1). (B) Protein expression was performed by Western blot in Human Synovial Sarcoma cell line (SW982), Human Knee Articular Chondrocytes cells (NHAC-Kn), Human Synoviocytes cells (HS), Human Dermal Fibroblast cells (NHDF-Ad), Human Epidermal Keratinocyte cells (NHEK-Ad), Human Dermal Microvascular Endothelial cells (HDMEC), Human Umbilical Vein Endothelium (HUVEC), Human Dermal Lymphatic Endothelial Cells (HDLEC) from affected compartments; focus on TIM1 receptor (n=1) and NPC1 receptor, which is expressed as mean ± SEM (n=2).

Supplementary figure 4

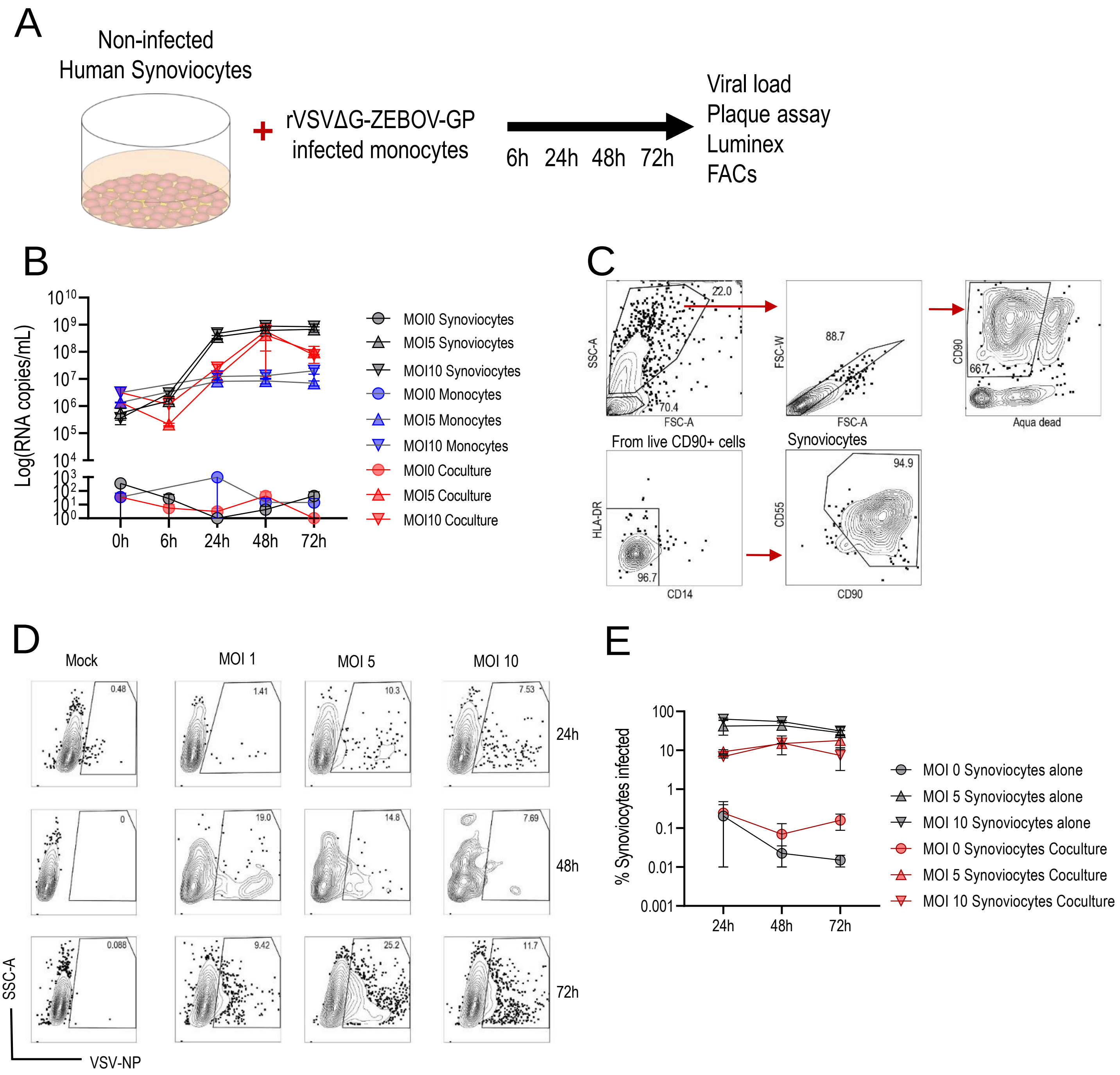

Supplementary figure 4: Co-culture sinoviocytes and infected monocytes (A). Scheme of the co-culture between non-infected Human Synoviocytes and infected CD14+ Monocytes. **(B)** Gate strategy for synoviocytes: morphological gate, live cells, CD14-, HLA-DR-, CD90+,CD55+ **(C)** FACS plots from gated synoviocytes, showing synoviocytes infected (VSV-NP+) from a representative sample at different time points after in-vitro infection (24h, 48h, 72h) and different viral inoculum (Mock, MOI 0, 5 and 10). The numbers indicate the percentage of the population in the previous gate. **(D).** Viral loads were measured in supernatant by RT-qPCR at 0h, 6h, 24h, 48h and 72h after in-vitro rVSV-ZEBOV infection (MOIs 0, 5 and 10) of synoviocytes (grey), monocytes (blue) or co-culture (red) and are expressed as mean ± SEM (n=2). **(E)** Percentage of infected Synoviocytes after in-vitro rVSV-ZEBOV infection (MOIs 0, 5 and 10) of synoviocytes (black) or co-culture synoviocytes (red). MOI 0 (circle), MOI 5 (triangle-up), MOI 10 (triangle-down).

Supplementary figure 5

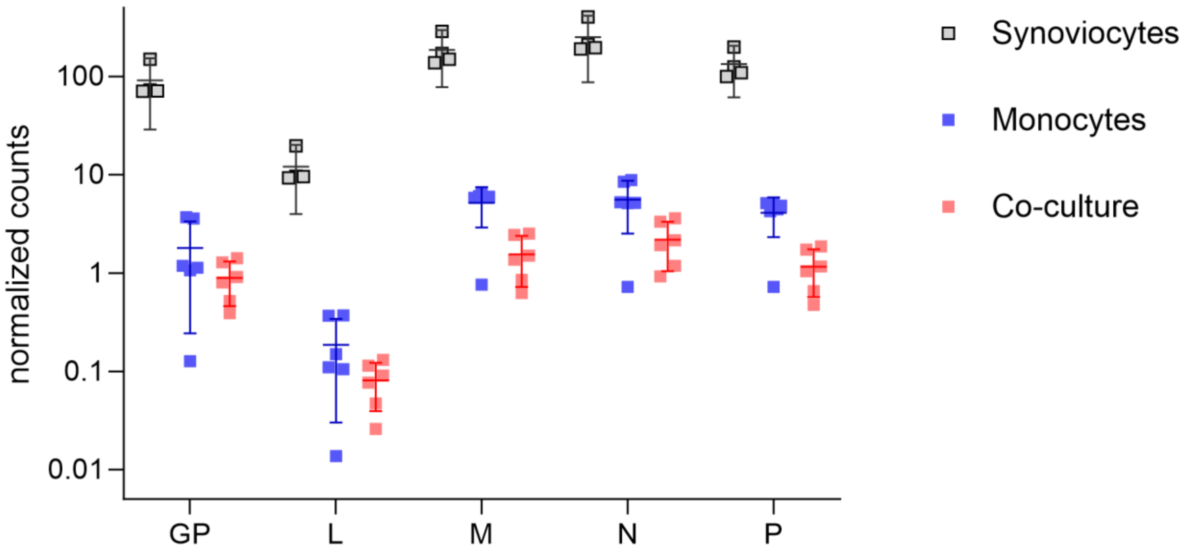

Supplementary figure 5: Normalized expression values of rVSVΔG-ZEBOV-GP in different cell cultures. Expression values were normalized by gene length, individual values are reported along with mean values and 95% confidence interval. GP=Ebola glycoprotein, L=large RNA-dependent RNA polymerase, M=Matrix protein, N=Nucleocapsid, P=phosphoprotein.

Supplementary figure 6

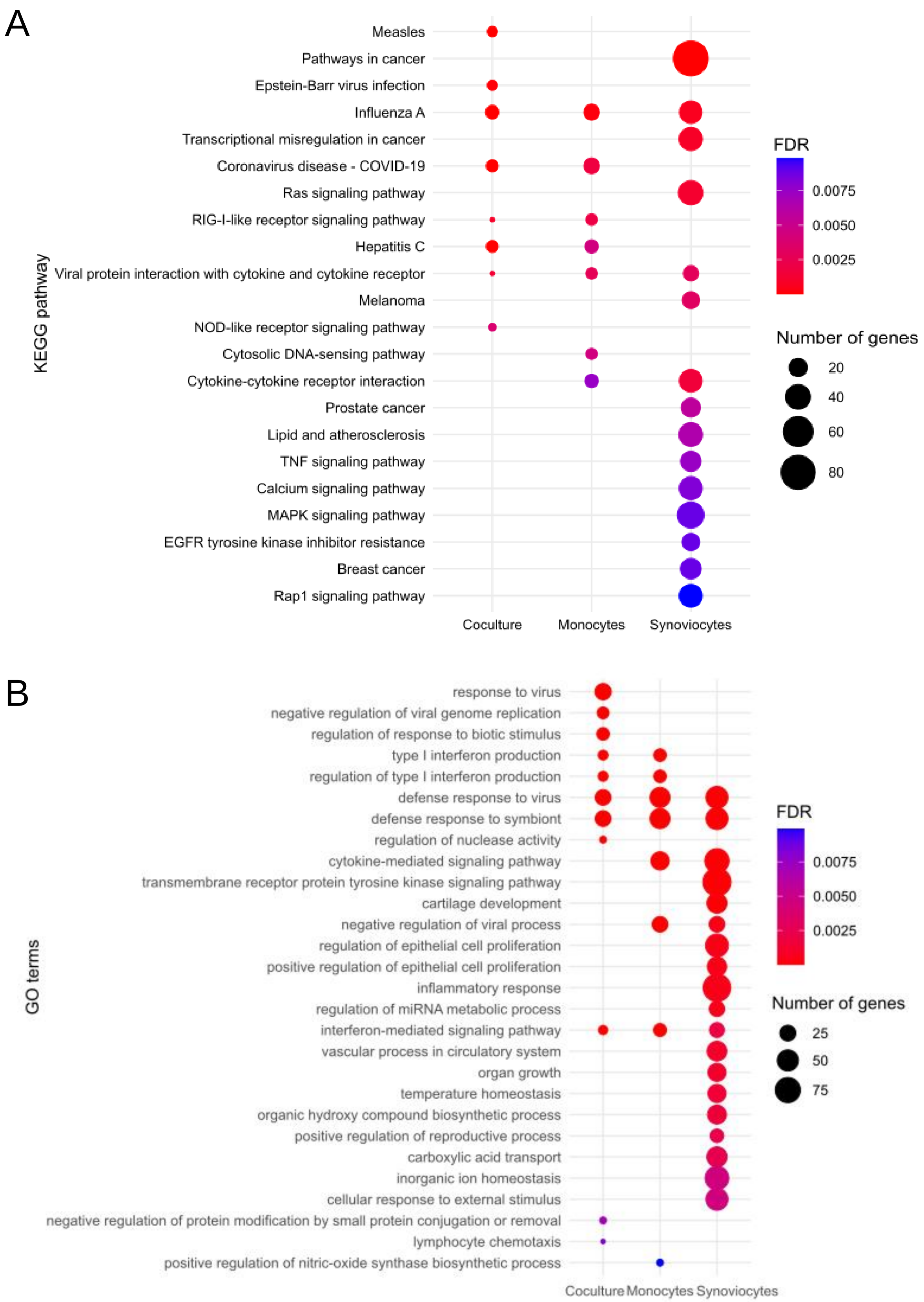

Supplementary figure 6: Activation of KEGG (A) and GO (B) pathways by rVSVΔG-ZEBOV-GP infection. Each column represents a cell line. Rows indicate different modules, which were significantly (FDR < 0.01) activated in at least one cell line. Each module is represented by a pie in which the significance of module activation is color scaled, while the effect size (i.e. the number of genes in the module) is proportional to its size.

Supplementary figure 7

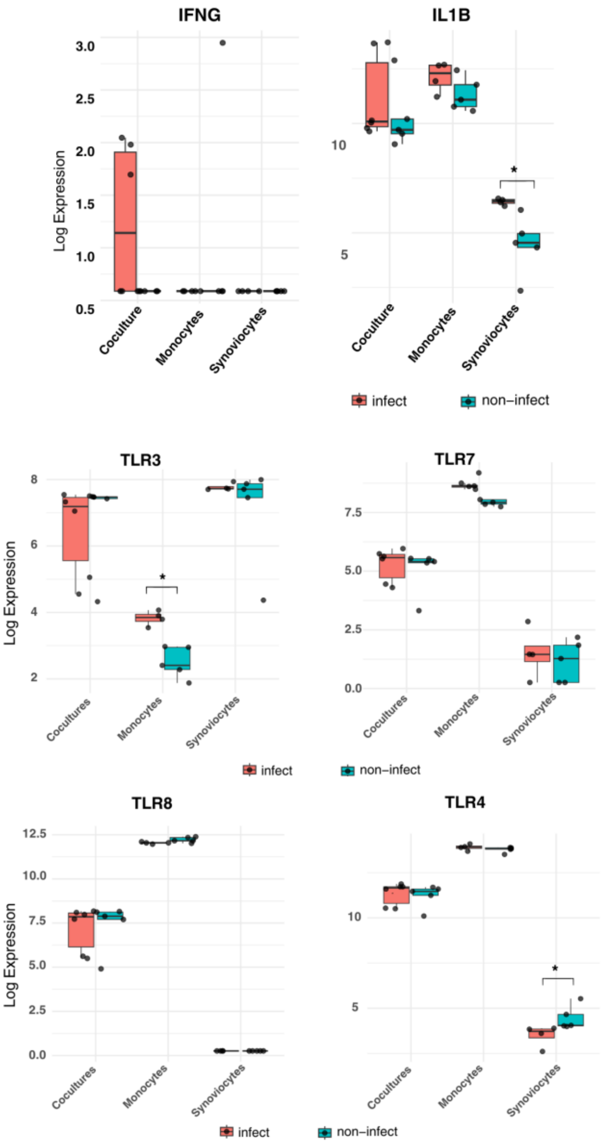

Supplementary figure 7: Normalized expression levels of genes coding for TLRs, IFNG and IL1B. Expression levels in infected (red) and uninfected (green) cell cultures of: IFNG, IL1B, TLR3, TLR4, TLR7, TLR9 genes. Data are reported as box and whiskers plot, where the marked line inside the box represented the median value, the box the interquartile range (IQR), and whiskers the minimum and maximum values in the range ±1.5*IQR. Individual values are reported as black dots. Differences in gene expression between infected and uninfected samples were assessed with the Wilcoxon test (* $p<0.05$, ** $p<0.01$).

Supplementary figure 8

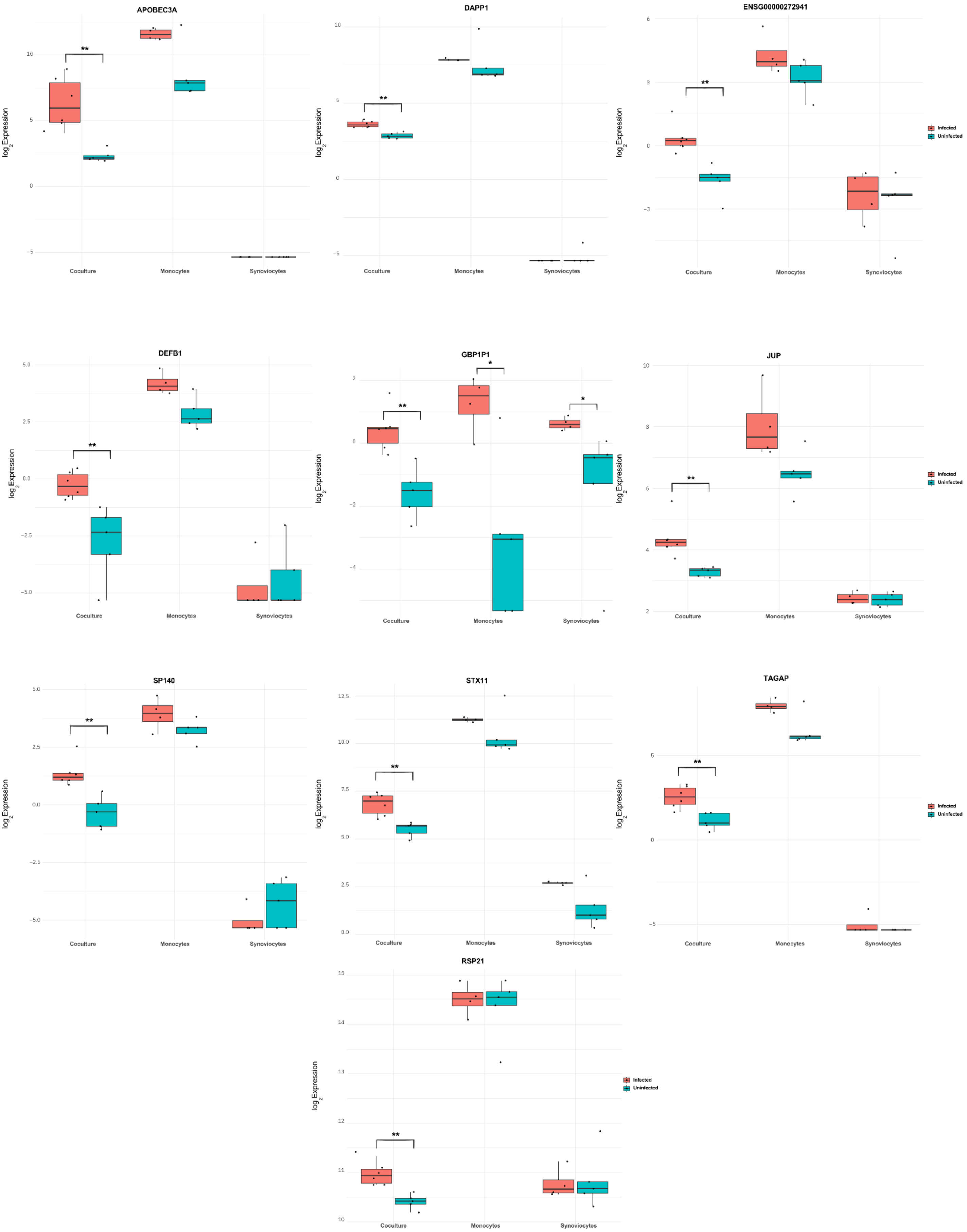

Supplementary figure 8: Normalized expression levels of genes uniquely DE in the coculture. Expression levels in infected and uninfected cell cultures of APOBEC3A, DAPP1, ENSG00000272941, DEFB1, GBP1P1, JUP, SP140, STX11, TAGAP and RSP21 genes. Data are reported as box and whiskers plot, where the marked line inside the box represented the median value, the box the interquartile range (IQR), and whiskers the minimum and maximum values in the range ±1.5*IQR. Individual values are reported as black dots. Differences in gene expression between infected and uninfected samples were assessed with the Wilcoxon test (** p<0.01, *** p<0.001).

Supplementary figure 9

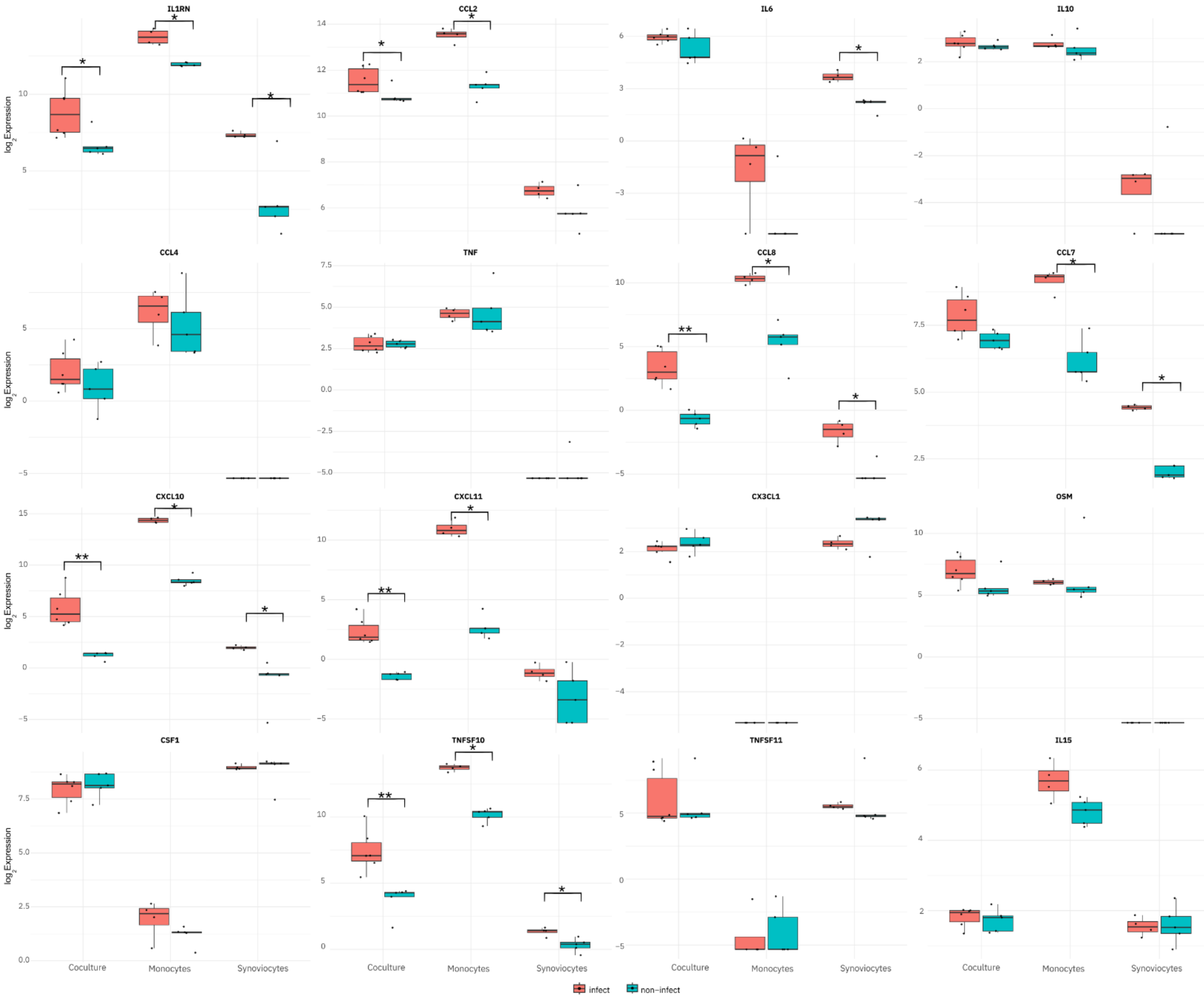

Supplementary figure 9: Normalized expression levels of genes coding for cytokines/chemokines. Expression levels in infected (red) and uninfected (green) cell cultures of: IL-1Ra, CCL2 (coding for MCP1), IL-6, IL-10, CCL4 (coding for MIP1b), TNF (coding for TNF-alpha), CCL8 (coding for MCP2), CCL7 (coding for MCP3), (coding for MCP4), CXCL10, CXCL11, CX3CL1, OSM, CSF1 (coding for MCSF), TNFSF10 (coding for TRAIL), TNFSF11 (coding for RANKL) and IL15 genes. Data are reported as box and whiskers plot, where the marked line inside the box represented the median value, the box the interquartile range (IQR), and whiskers the minimum and maximum values in the range ±1.5*IQR. Individual values are reported as black dots. Differences in gene expression between infected and uninfected samples were assessed with the Wilcoxon test (* $p<0.05$, ** $p<0.01$).

Supplementary table 1

Table S1: Overview of primary human cells and cell lines from the joint, skin and small vessels.

Tissue type	Name	Description
Joints	SW892	Human Synovial Sarcoma cell line
	HS	Human Synoviocytes
	NHAC-Kn	Human Knee Articular Chondrocytes
Skin	NHEK-Ad	Human Epidermal Keratinocytes
	NHDF-Ad	Human Dermal Fibroblasts
Vessels	HDMEC	Human Dermal Microvascular Endothelial Cells
	HDLEC	Human Dermal Lymphatic Endothelial Cells
	HUVEC	Human Umbilical Vein Endothelium

Supplementary table 2

Table S2: Number of differentially expressed genes after rVSV-ZEBOV infection in different cell lines.

	Up-regulated genes (%)	Down-regulated genes (%)	DE genes (%)	Unaffected genes	Expressed genes
**Monocytes**	208 (1.85)	40 (0.36)	248 (2.21)	10999	11247
**Synoviocytes**	848 (5.63)	876 (5.81)	1724 (11.44)	13344	15068
**Co-culture**	56 (0.36)	1 (0.0064)	57 (0.36)	15562	15619
